# Gastrointestinal perforation due to vasculitis at primary diagnosis of eosinophilic granulomatosis with polyangiitis (EGPA) despite a high dose glucocorticosteroids treatment

**DOI:** 10.1186/2193-1801-3-404

**Published:** 2014-08-04

**Authors:** Gunter Assmann, Marc Molinger, Michael Pfreundschuh, Rainer Bohle, Vincent Zimmer

**Affiliations:** University Medical School of Saarland, Rheumatology and Oncology, Kirrberger Strasse 1, D-66421 Homburg, Saar Germany; Department of Pathology, University Medical School of Saarland, Homburg, Saar Germany; Department of Medicine II Gastroenterology, University Medical School of Saarland, Homburg, Saar Germany

**Keywords:** Churg strauss, ANCA, Eosinophilic granulomatosis with polyangiitis, EGPA, Vasculitis, Gastrointestinal, Perforation

## Abstract

**Background:**

Eosinophilic granulomatosis with polyangiitis (EGPA) belongs to the systemic ANCA-associated vasculitides which may develop life-threatening major organ involvement, such as eosinophilic pulmonary infiltration, neuropathy, acute nephritis, myocarditis, and gastrointestinal (GI) tract involvement. Here, two cases of EGPA are presented developing perforation of the bowel at primary diagnosis after 7 respectively 10 days of initiation of high-dose glucocorticosteroides (GC) therapy.

**Findings:**

Two male patients, aged 32 (case 1) and 36 years (case 2), were admitted to the hospital with the symptoms of dyspnea, fatigue, fever, and chest pain. The patients completed the previously published revised international Chapel Hill nomenclature of EGPA. The two patients (case 1 and 2) developed acute abdominal pain after 7 (case 1) and ten days (case 2) on GC treatment. Computed tomography followed by surgery detected acute perforation of the small intestine (middle part of jejunum, case 1) and colon transversum (case 2). The resected specimens disclosed bowel perforation with severe transmural inflammation, edema, hemorrhage and vasculitis typically of EGPA. On the first post-operative day, therapy with cyclophosphamide (according to the Austin protocol) with a dosage of 750 mg/qm every 3 weeks (for 8 cycles) was initiated with good response within three months of treatment.

**Conclusion:**

The course of disease of the two presented EGPA patients suggest that very early initiation of intensified immunosuppressive treatment, preferentially with cyclophophomide or B cell depletion strategies, needs to be considered to avoid life-threatening complications of GI involvement.

## Introduction

Eosinophilic granulomatosis with polyangiitis (EGPA) belongs to the systemic vasculitides which may develop life-threatening major organ involvement Any type of vasculitis has the potential to cause local or diffuse pathological changes in the gastrointestinal (GI) tract based on the inflammation of vessel walls, followed by alterations of the blood flow and ischemic damage to the dependent organ (Çileda» et al. 2012; Singh et al. 2009; Venditti et al. 2011). Intestinal manifestations have to be considered more common in other vasculitis disease, such as polyarteriitis nodosa, however, it seems to occur as severe manifestation of EGPA (Nakamura et al. 2002). Several case reports have been published with EGPA and GI tract involvement. Venditti et al. have previously published a case of EGPA vasculopathy with large bowel perforation, however, the patient has been under long-term treatment of GC during his course of disease (Venditti et al. 2011). Two cases of EGPA have been presented with intestinal vasculitis, one of them with perforation as initial presentation of EGPA after 20 days of admission in the hospital (Venditti et al. 2011; Murakami et al. 2004). Varbanova et al. have presented a similar EGPA case to the cases, which here have been presented, showing vasculitic intestine perforation within 3 weeks after diagnosis of EGPA (Varbanova et al. 2011). Guellivin et al. have published data from a relatively large cohort of 96 patients: during the course of EGPA 30% of patients develop GI symptoms, such as abdominal pain, melena or hematemesis indicating intestinal vasulitic involvement (Guillevin et al. 1999). These data correspond to results from another cohort consisting in 150 EGPA patients previously published by (Moosig et al. 2013).

Here, two cases of EGPA are presented developing perforation of the bowel after initiation of high-dose GC therapy.

## Patients and methods

The study was performed in compliance with the Helsinki Declaration, all patients have given the informed written consent to take part in the study, the agreement to perform the study was given by the Ethic Committee Saarland.

Two male patients, aged 32 (case 1) and 36 years (case 2), were admitted to the hospital with the symptoms of dyspnea, fatigue, fever, and chest pain (Table 1). Following diagnostic procedures, the patients complied with the previously published revised international Chapel Hill nomenclature of EGPA (Jennette et al. 2013), showing major organ involvement: myocarditis with heart failure NYHA II, mononeuritis multiplex L1 (only case 1), polyneuropathy (only case 2), bronchial asthma with eosinophile infiltrations, mild proteinuria (only case 1), and glomerular hematuria (only case 2). After bronchoalveolar lavage and biopsy of the skin (only case 1), treatment with GC at a dose of 1 mg per kg/body weight was started. During GC administration the major organ manifestations were evaluated by completing diagnostic procedures, including biopsy of the suralis nerve (only case 1), endoscopy with multiple biopsies of the upper and lower intestine, MRI of the heart, and renal biopsy (only case 2).

**Table 1:** **Eosinophilic granulomatosis with polyangiitis patients’ characteristics (case 1, 2)**

Characteristics		Patient 1	Patient 2
**Time of diagnosis**		4/2010	1/2009
**Dosage of prednisolone** ^**a**^		80 mg	90 mg
**Days of GC treatment** ^**b**^		7	10
**Manifestations of EGPA**			
	**Heart failure (NYHA stage)**	II	II
	**Myocarditis** ^**c**^	Present	Present
	**Neuropathy**	Mononeuritis multiplex L1	Polyneuropathy
	**Lung**	Bronchial asthma	Asthma bronchiale
		Eosophile infiltration	
	**Kidney**	Mild proteinuria (0.5 g/24 h)	Glomerular hematuria
	**Eosinophilic granulocytes** ^**d**^	5500/μl	4900/μl
**IgE value**		Elevated	Elevated
**pANCA value**	**(Positive)** ^**e**^	12.8 U/ml^f^	14.1 U/ml^f^

^a^intraveneous application once a day; ^b^GC = glucocorticosteroides, till perforation followed by abdominal surgery; EGPA = eosinophilic granulomatosis with polyangiitis; ^c^histological proved by myocardial biopsy; ^d^counted in periphereal blood; ^e^pANCA = perinuclear pattern of antineutrophilic cytoplasmatic autoantibodies, tested by immunofluorescence test and by ELISA for myeloperoxidase autoantibodies (anti-MPO) ^f^in U/ml; NYHA = New York Heart Association classification of heart failure.

## Results

**Figure 1:**
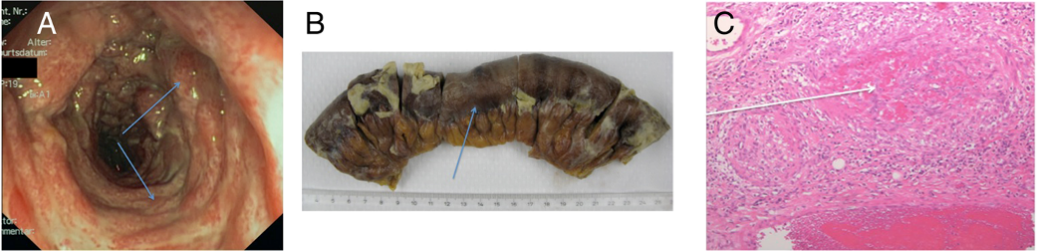
**Colon transversum perforation of Churg Strauss patient (case 2) due to vasculitis. A**: EGPA in case 2 and endoscopy in transverse colon with vasculitis lesions. **B**: EGPA in case 2 and macroscopic view of the resected transverse colon with vasculitis necrotizing lesions after surgery. **C**: EGPA in case 2 and histologic preparation of the resected transvere colon with eosinophilic infiltration and thrombotic vessel occlusion.

The two patients (case 1 and 2) developed acute abdominal pain after 7 (case 1) and ten days (case 2) on GC treatment and three and five days, respectively, after endoscopy. The performed biopsies during the endoscopy demonstrated vasculitis spots in both patients, but only in the upper intestine. Computed tomography followed by surgery detected an acute perforation of the small intestine (middle part of jejunum, case 1) and colon transversum (case 2). The resected specimen disclosed bowel perforation with severe transmural inflammation, edema, hemorrhage and vasculitis typical of EGPA. Figure 1 shows the endoscopic (figure A) and macroscopic view (figure B) of the resection specimen with multiple vasculitis induced necrotizing lesions with the corresponding histological preparation (figure C) after surgery (case 2). The colon perforation in case two was shown not to have occurred due to previously performed endoscopy biopsies. On the first post-operative day, therapy with cyclophosphamide due to major organ involvement (according to the Austin protocol) with a dose of 750 mg/qm every 3 weeks (for 8 cycles) was initiated with good response within three months of treatment (case 1 and 2). Until the sixth month, the patients received azathioprine 2.5 mg/kg body weight without relapse of disease, and a reduction to a dose of 7.5 mg prednisolone per day was implemented.

The good response of treatment was documented in reduction of eoasinophilic granulocytes lower than 2000/μl, normalization of the lung spirometry, complete remission of the myocarditis (measeared with MRI), and normalization of kidney function (with normal urine sediment test). However, both of them showed not ameliorated but persistent neuropathy, and patient one suffered from a persistent reduced ejection fraction of the heart after 12 weeks. The p-ANCA levels have not changed during the treatment period in both cases.

## Discussion

We present two cases of GI perforation within two weeks after primary diagnosis of EGPA under treatment of high-dose GC. The macroscopic and histologic preparation of the involved intestinal tract proved unconstrained inflammation as the most probable cause of perforation. After immediate postoperative initiation of intravenous cyclophosphamide treatment both cases have shown clinical remission during the following 12 weeks.

The treatment of EGPA with GI vasculitic involvement at primary diagnosis may not be sufficiently treated by GC alone to protect from life-threatening and very early occuring complications such as GI perforation. Murakami et al. has published a similar case of primary diagnosis of EGPA with GI perforation 20 days after admission to hospital, however, the GC dosage was lower (Murakami et al. 2004).

In general, GI involvement seems to be an indicator of poor prognosis in this disease entity and has been reported in an estimated 20 – 50% of patients (Guillevin et al. 1996; Hayami et al. 2012; Bourgarit et al. 2005). However, larger, more informative clinical study cohorts of EGPA patients to evaluate the true prognostic impact of GI vasculitis are currently not available. Nevertheless, previously published data from the French Vasculitis Study Group have evaluated the “*five factors score*” (FFS) to predict the risk of death due to EGPA (Bourgarit et al. 2005); the FFS score is widely used to assess the EGPA prognosis by giving one point for the involvement of GI, one point for involvement of the heart and the central nervous system, respectively, and one or two for the kidney manifestation (depending on serum creatinine and proteinuria) (Pagnoux et al. 2007; Guillevin et al. 2011; Vaglio et al. 2012). In general, Moosig et al. has demonstrated the probable benefit of immunosuppressive treatment beyond GC in all stages of EGPA patients out of the Northern German vasculitis centre cohort (Moosig et al. 2013); in detail, with an initial FFS (score) of one or higher, the intravenous bolus application of cyclophosphamide has been documented as the most frequent treatment regime (71%).

In synopsis with the two cases we have presented here, there appears to be a rationale for an intensified immunosuppressive treatment approach beyond GC within the first week after EGPA diagnosis with GI involvement; conventional therapies such as cyclophosphasmide or previously establishing biological treatment modalities such as B cell depletion needs to be considered to avoid life-threatening GI manifestations with the goal to improve the outcome.
